# Medicines and Meddlesomeness.—II

**Published:** 1922-11

**Authors:** R. W. Harris

**Affiliations:** (formerly Assistant Secretary, responsible for the administraton of National Health Insurance Medical Benefits, Ministry of Health)


### MEDICINES AND MEDDLESOMENESS.—II.

**By R. W. HARRIS (formerly Assistant Secretary, responsible for the administration of National Health Insurance Medical Benefits, Ministry of Health).**

THE Panel Committees, who are entrusted with the task of investigating whether the cost of the medicines supplied to a doctor's insurance patients is "in excess of what may reasonably be necessary for their adequate treatment," are given a practical task, for which, as medical men, usually in general practice, they are well qualified. It is fair to say that those who criticise this system, as imposing an unfair task on the Panel Committee, include others besides meddlers, although, of course, the latter are the most vocal. The answer to this criticism, in the first place, is that the task was imposed on the medical profession at their own request, and, in the second place, that the very delicacy of the task itself ought to be such, on the one hand, as to stimulate the Panel Committee to discharge it thoroughly as an honourable obligation, and to cause a practitioner, the cost of whose prescribing is called into question, to appreciate that the men conducting the investigation are ready and anxious to give him a thoroughly fair hearing. The task, I say, is a practical one, involving a combination of the judicial function with the exercise of common-sense—two things which ought to be, but are not always, concurrent.

In order to assist the Panel Committees in their task, which would otherwise involve the yearly handling in most areas of tens of thousands of prescriptions, and, in some areas, hundreds of thousands, it was felt to be essential that the material which they are called upon from time to time to bring under review should be properly tabulated, and also carefully limited in amount. With this object, the bureaux which carry out the work of pricing chemists' prescriptions (which, for the whole country, number over 30,000,000 per annum) send from time to time to the Panel Committees an analysis of the prescribing of doctors whose total cost of prescribing is beyond a certain datum line, that line being drawn at an amount 20, 30, 40 (or some other figure) per cent. in excess of the average cost of prescribing for the area. The datum line is fixed locally, and the sole object is to ensure that the Panel Committee is not presented with an unwieldy mass of material for examination. It is here that the meddler takes another jump, this time leaping to the conclusion that there will be an automatic surcharge on a doctor because the cost of his prescribing has passed the datum line. The fault that I find with this sort of irresponsible critic is that he does not credit those responsible for the system with even a small dose of common-sense, and it may not occur to him that, in his condemnation of the system, he involves not merely the public servant, but the representatives of the medical profession, who have been called into consultation before arrangements of this sort are made.

Many decisions of the Ministry of Health have been published, a review of which indicates that the Department recognises to the full that, in considering the cost of a doctor's prescribing, account must be taken of the facts of his practice; of such questions as to whether his patients, considered as a whole, require more than the usual amount of treatment; whether he has an abnormal number of patients of a particular class—*e.g.*, elderly or chronic patients, or patients from works where some diseases or accidents are particularly prevalent. It is further recognised in such decisions that it is difficult to apply the law of averages satisfactorily where the number of patients is small; and especially is it recognised that reasonable allowance must be made for the personal element in prescribing, for the exercise of initiative and for suitable experiment. The Department have stated that the enforcement of stereotyped methods of standards of prescribing would be prejudicial both to the immediate interest of patients under treatment, and to the ultimate interest of insured persons generally. On the other hand, it is clear that doctors, in looking through the prescriptions of one of their own number where the total cost is greatly in excess of the general cost, would have little difficulty in determining whether there was extravagance, and where the extravagance would appear to lie. The heavy cost may be due to the use of expensive preparations where others of clearly the same therapeutic value are available at less cost, or to the unnecessary frequency with which prescriptions are written for the same patient—*e.g.*, the ordering of bottles of medicines or supplies of pills before the last supply can possibly have been exhausted, if properly used; or it may arise from the continuance of supplies of drugs for an exceptionally long period, or from other forms of carelessness or indifference to the question of economy.

It is as well to emphasise that all these matters are questions for inquiry only in the first instance, and that a doctor can often dispose of them with an adequate explanation. It would, of course, be out of the question that the Panel Committee should be saddled with the task of demonstrating in the case of each individual patient that there had been extravagance. A nimble-witted doctor might be ready with an explanation in each case which the Panel Committee could not possibly controvert, as the doctor alone can know the circumstances of his individual patients. But, as stated in one of the decisions of the Department, "where there has been such an exceptional incidence of cases of a particular kind as seriously to affect the average, it is difficult to believe that that fact and the causes of abnormality will not be apparent and easily demonstrated."

The announcement which has been made that the Regional Medical Staff will be associated in future with the Insurance Committees and Panel Committees in the investigation of questions of alleged excessive prescribing will undoubtedly give the meddlers another opening. We shall be assured that the representatives of the insurance practitioners themselves, the practical men, the men on the spot, are to be ousted by the Minister's minions from Whitehall. I think that it will be found that in the work of preliminary investigation of the mass of material contained in the statistics of prescriptions thrown up by the pricing bureaux there is a good deal of solid work of which Panel Committees will be glad to be relieved, if they know that the prescriptions are being scrutinised by competent medical officers, and that this will help to clear out of the way many cases which are readily susceptible of simple explanation without the uncomfortable formality and waste of time involved in the doctor's appearance at a hearing by the Panel Committee. It will also be found that the Minister's minions will not encroach on the judicial functions of the Panel Committees.

An interesting suggestion, referred to in another column, was made in a paper read at the recent Conference of Clerks to Insurance Committees by the Clerk to the Coventry Insurance Committee, Mr. Lee Gordon. It is to the effect that a specially constituted sub-committee of the Insurance Committee, with an assured medical majority and a medical chairman, should have transferred to it the functions in regard to investigation of prescribing now performed by Panel Committees. It may not find support in medical circles generally, but it at least merits the careful consideration of all who are interested in this question. Mr. Lee Gordon misconceives, in my view, the intentions of the Department with regard to the Regional Medical Staff, and he has misapprehended the lines of a decision which he seems to think has discouraged some Panel Committees from a proper activity. In so far as he bases his proposals for a change of machinery on these two considerations, he is not, I think, on very secure ground. But his paper is a thoughtful contribution to a subject full of difficulty, and I hasten to add that I can find in it no trace of meddlesomeness.

